# Risk Factors and Prognostic Models in Acute Large Vessel Occlusion Stroke: Insights From ASPECTS‐Net Water Uptake

**DOI:** 10.1002/brb3.70544

**Published:** 2025-05-13

**Authors:** Hongru Ou, Huanhua Wu, Shuolong Wu, Qian Cao, Xiaozheng Cao, Guanye Zhang, Jiabin Mo, Youzhu Hu

**Affiliations:** ^1^ Department of Radiology The Affiliated Shunde Hospital of Jinan University Foshan Guangdong P. R. China; ^2^ Central Laboratory The Affiliated Shunde Hospital of Jinan University Foshan Guangdong P. R. China; ^3^ Department of Neurosurgery The Affiliated Shunde Hospital of Jinan University Foshan Guangdong P. R. China; ^4^ Department of General Surgery The Affiliated Shunde Hospital of Jinan University Foshan Guangdong P. R. China; ^5^ Department of Hepatobiliary Surgery The First Affiliated Hospital Jinan University Guangzhou Guangdong P. R. China

**Keywords:** acute ischemic stroke, Alberta Stroke Program Early CT Score (ASPECTS), net water uptake, nomogram, prognosis

## Abstract

**Background:**

The outcomes of endovascular reperfusion in acute large vessel occlusion stroke (ALVOS) vary, with some patients recovering fully while others face disability or mortality despite recanalization. Alberta stroke program early CT score‐net water uptake (ASPECTS‐NWU), a quantitative imaging metric assessing tissue edema and infarct progression, may improve prognostic accuracy.

**Methods:**

This study included 96 ALVOS patients between December 2020 and March 2024. Patients were categorized into good (modified Rankin Scale [mRS] 0–2) and poor (mRS 3–6) prognosis groups based on 90‐day mRS outcomes. Feature selection using Least Absolute Shrinkage and Selection Operator (LASSO), Boruta, and logistic regression (LR) identified key predictors, including LVO, Alberta Stroke Program Early CT Score (ASPECTS), ASPECTS from the follow‐up CT (ASPECTSFCT), and National Institutes of Health Stroke Scale (NIHSS) scores. Predictive performance was validated with cross‐validation, and model calibration was assessed via calibration curves, receiver operating characteristic (ROC) curves, area under the curve (AUC), and the Spiegelhalter *Z*‐test (significance set at *p* < 0.05).

**Results:**

LR highlighted LVO, ASPECTS, ASPECTSFCT, and NIHSS as significant predictors of poor prognosis. The constructed nomogram enables individualized risk assessment, with total points correlating to poor outcome probability. ROC analysis showed good discriminatory ability in the training set (AUC 0.815, 95% confidence interval [CI]: 0.714–0.916) but moderate performance in the test set (AUC 0.688, 95% CI: 0.484–0.891). Calibration was strong in the training set (Spiegelhalter *Z* < 0.0001) but showed minor issues in the test set (Spiegelhalter *Z* = 1.222).

**Conclusions:**

This study highlights the prognostic value of ASPECTS‐NWU in ALVOS and its integration into a predictive nomogram for individualized risk assessment. By refining ischemic injury stratification, ASPECTS‐NWU can guide therapeutic decisions, optimize post‐reperfusion management, and improve patient outcomes.

## Graphical abstract

Our analysis identifies significant predictors of poor prognosis in anterior circulation acute large vessel occlusive stroke (ALVOS) patients after endovascular reperfusion. Specifically, ASPECTS‐net water uptake (ASPECTS‐NWU), combined with NIHSS and large vessel occlusion (LVO), demonstrates strong prognostic value. These findings highlight ASPECTS‐NWU as a promising biomarker for individualized risk assessment and provide insights into optimizing post‐reperfusion outcomes in ALVOS patients.

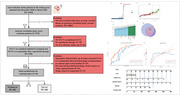

**Abbreviations**
AIS: acute ischemic stroke
ALVOS: anterior circulation acute large vessel occlusive stroke
ASPECTS: Alberta stroke program early CT score
AUC: area under the curve
CIs: confidence intervals
HU: Hounsfield unit
LASSO: Least Absolute Shrinkage and Selection Operator
LR: logistic regression
mRS: modified Rankin scale
NIHSS: National Institutes of Health Stroke Scale
NWU: net water uptake
ROC: receiver operating characteristic
ROI: region of interest

## 1 Introduction

Acute ischemic stroke (AIS) remains a major global health burden, with anterior circulation acute large vessel occlusion stroke (ALVOS) carrying particularly high risks of severe neurological impairment and mortality (Feigin et al. 2019; Powers 2020). Despite advances in endovascular reperfusion therapy, a significant proportion of ALVOS patients experience poor functional outcomes, even after successful recanalization (Dekker et al. 2023). Studies indicate that up to 50% of patients fail to achieve functional independence post‐reperfusion, underscoring the need for more precise prognostic tools to guide early clinical decisions (Baron 2018; Wang et al. 2024). Accurate early prognosis is essential for optimizing patient management, as it enables timely risk stratification, prioritization of intensive monitoring, and allocation of rehabilitation resources (Phipps and Cronin 2020).

The Alberta Stroke Program Early CT Score (ASPECTS) is widely used to assess early ischemic changes in acute stroke (Baig et al. 2023). Although ASPECTS provides valuable structural insights, it does not capture dynamic ischemic progression or edema formation, both of which significantly impact patient outcomes (Broocks et al. 2020; Teo et al. 2021). ASPECTS‐net water uptake (ASPECTS‐NWU) refines this approach by quantifying ischemic edema, offering a more comprehensive assessment of tissue viability (Broocks et al. 2024). By measuring water uptake differences between ischemic and non‐ischemic brain regions, ASPECTS‐NWU provides a more precise indicator of infarct progression, improving risk prediction and guiding treatment decisions in ALVOS patients (Lu et al. 2022).

Despite advancements in stroke imaging, existing prognostic models lack sufficient accuracy for individualized risk prediction (Ballout et al. 2024). ASPECTS primarily evaluates infarct extent, whereas widely used clinical scales such as the National Institutes of Health Stroke Scale (NIHSS) provide only a general severity grading, failing to specifically identify high‐risk patients requiring early intervention (Saito et al. 2020). Although multimodal imaging markers have been explored to improve risk stratification, many current models rely on qualitative or semi‐quantitative assessments, leading to inconsistencies and interobserver variability (Czap and Sheth 2021; Jiang et al. 2024). To address these gaps, we propose an ASPECTS‐NWU‐based nomogram that integrates key imaging and clinical predictors into an objective, quantitative risk model. This tool aims to enhance individualized risk assessment, improve patient stratification, and facilitate timely therapeutic decision‐making. By identifying high‐risk patients early, the nomogram may support proactive intervention, optimize resource allocation, and ultimately improve post‐reperfusion outcomes.

Although the prior research has established that factors, such as LVO, baseline ASPECTS, and NIHSS scores, significantly correlate with ALVOS prognosis (Singh et al. 2023; Al‐Mufti et al. 2018), an integrated predictive model incorporating quantitative ischemic edema metrics remains lacking. Therefore, this study aims to evaluate the prognostic value of ASPECTS‐NWU, identify key risk factors for poor outcomes, and develop a predictive nomogram that outperforms existing models in ALVOS prognosis.

## 2 Materials and Methods

### 2.1 Study Design and Patients

This retrospective study was conducted at The Affiliated Shunde Hospital of Jinan University, focusing on patients diagnosed with ALVOS who underwent reperfusion treatment between December 2020 and March 2024. The study protocol was approved by the hospital's ethics committee, and informed consent was obtained from all patients or, in cases where patients were unable to provide consent, from their legal representatives.

**FIGURE 1:**
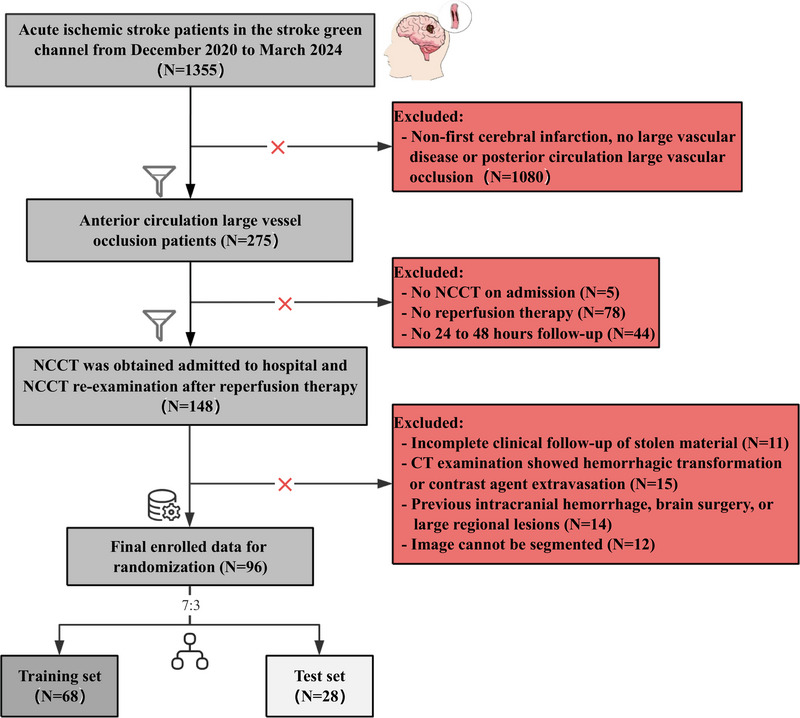
Patient selection flowchart illustrating the selection process of acute ischemic stroke patients. NCCT, non‐contrast CT.

Inclusion criteria comprised adults aged 18 years or older with anterior circulation stroke and confirmed LVO via CT or MRI. Patients were classified into two groups based on 90‐day functional outcomes using the modified Rankin Scale (mRS): the good prognosis (mRS 0–2) and the poor prognosis (mRS 3–6) (Broderick et al. 2017). As illustrated in Figure 1, the study followed a structured patient selection process. Of 1355 AIS patients initially screened, 275 met criteria for anterior circulation LVO. After applying additional exclusion criteria—including absence of non‐contrast CT (NCCT) on admission, lack of reperfusion therapy, incomplete follow‐up, hemorrhagic transformation, or pre‐existing intracranial pathology—96 patients were included in the final analysis. These patients were then randomized into a training set (*N* = 68) and a test set (*N* = 28) using a 7:3 ratio. The final cohort's demographic data, clinical characteristics, and imaging findings were systematically collected, including baseline NIHSS scores, ASPECTS, ASPECTSFCT, and LVO presence, which were analyzed to identify significant prognostic factors. The study employed a rigorous feature selection process utilizing Least Absolute Shrinkage and Selection Operator (LASSO), Boruta, and logistic regression (LR) to determine the most relevant predictors of poor prognosis in ALVOS patients.

### 2.2 Brain CT Examination

Brain CT examinations were conducted using a Siemens 64‐slice 128‐layer spiral CT scanner (SOMATOM Definition AS + 64 Slice). Initial NCCT scans were performed to exclude hemorrhagic events and brain tumors. The scanning parameters for NCCT were set at 120 kV, 420 mA, with a slice thickness and interval of 5 mm. For further evaluation, CT angiography (CTA) of the head and neck was performed, covering the area from the tracheal bifurcation to the skull vertex. This included two imaging phases, followed by vascular enhancement imaging. Enhancement monitoring was initiated at the tracheal bifurcation level, with the region of interest (ROI) placed at the descending aorta. The dynamic monitoring CT value of the ROI was recorded using an automated injection tracking system.

A 50 mL dose of non‐ionic iodinated contrast agent (iohexol, 370 mgI/mL) was administered via a high‐pressure injector through the right antecubital vein, followed by a 40 mL isotonic saline flush at 3.5 mL/s. Monitoring began 10 s post‐injection of the contrast agent, with a trigger threshold set at 100 HU. Scanning was initiated immediately upon reaching the threshold. For CTA, the scanning parameters included a tube voltage of 120 kV, with the tube current automatically adjusted using Care Dose 4D technology to optimize radiation exposure. Additional parameters were a pitch of 1.1, collimation of 128 × 0.6 mm, rotation time of 0.33 s, and a slice thickness of 0.6 mm.

### 2.3 Endpoints and Definitions

The primary endpoints of this study included the efficacy of intravenous thrombolysis with alteplase and the neurological outcomes assessed at 90 days post‐treatment. Intravenous thrombolysis was chosen as the primary focus because it is the standard first‐line reperfusion therapy for AIS, often preceding or complementing endovascular treatment (Widimsky et al. 2023). Evaluating its efficacy provides insight into early treatment response, which is crucial for guiding acute stroke management strategies. All patients in this study were eligible for intravenous thrombolysis with alteplase, which was administered as the primary reperfusion treatment within the designated treatment window. However, in cases where patients were ineligible for endovascular treatment or declined the procedure, conservative medical management was implemented. Conservative management consisted of dual antiplatelet therapy combined with high‐intensity statin therapy, aimed at secondary stroke prevention and optimizing medical stabilization. Although these patients did not undergo endovascular intervention, their neurological outcomes were still assessed alongside the primary treatment group to evaluate prognostic factors across different therapeutic approaches.

Neurological function was assessed using the NIHSS at baseline by the attending physician. At 90 days post‐treatment, neurological evaluations were conducted via outpatient follow‐ups or telephone consultations. The mRS was used to categorize patient outcomes, with a score of ≤2 indicating favorable outcomes, whereas a score of > 2 reflected varying degrees of disability, ranging from moderate functional impairment to severe disability or death.

### 2.4 Calculation of ASPECTS‐NWU

The ASPECTS‐NWU was evaluated through a comprehensive imaging analysis utilizing an automated ASPECT scoring system software (SHUKUN‐ASPECTS). This software calculated the average Hounsfield unit (HU) values for each defined ROI on the CT scans, providing an objective assessment of ischemic tissue characteristics.

The use of HU values is well‐established in stroke imaging, as it reflects tissue density changes associated with infarction and edema. To derive ASPECTS‐NWU values, HU measurements were taken from both the ischemic region (HU_ischemic) and the contralateral normal region (HU_normal), with their relative difference expressed as a percentage to represent the extent of NWU. This approach is particularly relevant because HU attenuation differences correlate with ischemic edema severity, making them a reliable marker for infarct progression; normalization using the contralateral normal region accounts for interpatient variability in baseline CT attenuation, improving consistency across measurements; and quantifying NWU enables a more precise evaluation of early ischemic damage and its potential impact on post‐reperfusion outcomes. The formula used for calculating ASPECTS‐NWU% is as follows:

(1) $$\mathrm{ASPECTS}-\mathrm{NWU}\%=\left[1-\left(\frac{HU_{\mathrm{ischemic}}}{HU_{\mathrm{normal}}}\right)\right]\times 100$$

In addition to the initial ASPECTS‐NWU calculation at admission, follow‐up CT scans were performed to monitor ischemic edema progression and assess dynamic changes in infarct evolution post‐reperfusion. Given that edema formation is a key determinant of secondary brain injury, the change in NWU (ΔNWU) over time provides valuable insight into the effectiveness of reperfusion therapy and the risk of further neurological deterioration. The progression of lesion edema was quantified using ΔNWU, defined as the difference between the ASPECTS‐NWU from the follow‐up CT (ASPECTS‐NWUFCT) and the admission CT (ASPECTS‐NWU admission). The formula for ΔNWU is expressed as follows:

(2) $$\Delta \mathrm{NWU}=\mathrm{NW}U_{\mathrm{FCT}}-\mathrm{NW}U_{\mathrm{admission}}$$

### 2.5 Statistical Analysis

Statistical analyses were conducted using R software, version 4.3.1 (packages meta, R foundation). Descriptive statistics were calculated, with continuous variables reported as means ± standard deviations (SD) or medians (interquartile ranges [IQR]), depending on data distribution, and categorical variables presented as frequencies and percentages. Group comparisons based on 90‐day outcomes were performed using *t*‐tests or Mann–Whitney *U* tests for continuous variables, depending on normality, and Chi‐square tests or Fisher's exact tests for categorical variables, as appropriate.

Feature selection for predictive modeling was performed using LASSO regression, Boruta, and LR, each chosen for its distinct strengths in identifying key prognostic variables (Fahimifar et al. 2023). LASSO regression was applied to handle high‐dimensional data and mitigate multicollinearity by shrinking less relevant coefficients to zero, ensuring retention of only the most predictive variables (Wu et al. 2024). Boruta, a feature selection algorithm based on Random Forest, identified the most relevant predictors by iteratively comparing them to randomized shadow variables, enhancing robustness against noise and redundancy (Liao et al. 2024). LR was selected for its ability to construct an interpretable predictive model with well‐established performance in binary classification tasks. The performance of the LR model was evaluated through *k*‐fold cross‐validation techniques to ensure robustness. Calibration of the model was assessed with calibration curves, whereas the discriminatory ability was measured using receiver operating characteristic (ROC) curves, with the area under the curve (AUC) calculated alongside 95% confidence intervals (CIs). Statistical significance was set at a threshold of *p* < 0.05.

## 3 Results

### 3.1 Demographic and Clinical Data

A total of 96 patients diagnosed with ALVOS were included in the study. The demographic and clinical characteristics of the study population, stratified by mRS outcomes, are summarized in Table 1.

**TABLE 1:** Baseline characteristics by modified Rankin Scale (mRS) group (good vs. poor) in the training and test sets.

Variable^a^	Training set (*N* = 68)			Test set (*N* = 28)		
	Good (*N* = 41)	Poor (*N* = 27)	*p* value^b^	Good (*N* = 16)	Poor (*n* = 12)	*p* value^b^
**ASPECTS.NWU (mean ± SD)**	2.1 ± 1.6	2.5 ± 1.8	0.374	1.3 ± 1.2	3.0 ± 3.3	0.114
**ASPECTS (mean** ± **SD)**	9.5 ± 1.1	8.2 ± 2.1	0.005	9.4 ± 1.0	8.8 ± 2.8	0.435
**TIME, h (mean** ± **SD)**	21.7 ± 6.0	23.4 ± 9.0	0.373	22.7 ± 6.2	16.6 ± 8.2	0.034
**ASPECTSFCT (mean** ± **SD)**	8.4 ± 1.8	5.6 ± 3.6	< 0.001	8.1 ± 2.4	6.9 ± 3.1	0.256
**ΔASPECT (mean** ± **SD)**	1.1 ± 1.7	2.6 ± 3.7	0.065	1.3 ± 2.1	1.8 ± 2.4	0.543
**ASPECTS.NWUFCT (mean** ± **SD)**	2.8 ± 2.0	5.9 ± 4.6	0.002	2.2 ± 2.7	4.1 ± 4.9	0.239
**CTT, min (mean** ± **SD)**	105.3 ± 81.8	76.8 ± 87.4	0.176	73.9 ± 49.1	91.3 ± 63.0	0.418
**TO2I, h (mean** ± **SD)**	1.9 ± 1.3	1.4 ± 1.4	0.186	1.5 ± 0.9	1.5 ± 1.0	0.888
**ΔNWU (mean** ± **SD)**	0.6 ± 2.0	3.4 ± 4.7	0.007	0.9 ± 2.0	1.1 ± 2.9	0.787
**ASPECTS.NWU.time (mean** ± **SD)**	0.0 ± 0.1	0.2 ± 0.3	0.048	0.1 ± 0.2	0.1 ± 0.2	0.743
**ASPECTS.NWU.log.time.1 (mean** ± **SD)**	0.5 ± 1.5	2.5 ± 3.7	0.012	0.7 ± 1.7	0.9 ± 2.3	0.775
**LVO**			0.033			0.669
MCA	29 (70.7%)	11 (40.7%)		14 (87.5%)	11 (91.7%)	
ICA	2 (4.9%)	1 (3.7%)		1 (6.2%)	0 (0%)	
MCA and ICA	10 (24.4%)	15 (55.6%)		1 (6.2%)	1 (8.3%)	
**IAT**			0.687			0.770
No	21 (51.2%)	16 (59.3%)		10 (62.5%)	9 (75%)	
Yes	20 (48.8%)	11 (40.7%)		6 (37.5%)	3 (25%)	
**Gender**			0.768			0.956
Male	27 (65.9%)	16 (59.3%)		8 (50%)	7 (58.3%)	
Female	14 (34.1%)	11 (40.7%)		8 (50%)	5 (41.7%)	
**Age**	62.1 ± 13.2	70.0 ± 14.1	.023	63.6 ± 10.1	70.3 ± 13.8	0.144
**Elder**			0.035			0.391
No	38 (92.7%)	19 (70.4%)		15 (93.8%)	9 (75%)	
Yes	3 (7.3%)	8 (29.6%)		1 (6.2%)	3 (25%)	
**HTN**			0.246			0.620
No	10 (24.4%)	11 (40.7%)		8 (50%)	4 (33.3%)	
Yes	31 (75.6%)	16 (59.3%)		8 (50%)	8 (66.7%)	
**DM**			1.000			1.000
No	34 (82.9%)	22 (81.5%)		13 (81.2%)	10 (83.3%)	
Yes	7 (17.1%)	5 (18.5%)		3 (18.8%)	2 (16.7%)	
**AF**			0.128			0.539
No	35 (85.4%)	18 (66.7%)		11 (68.8%)	6 (50%)	
Yes	6 (14.6%)	9 (33.3%)		5 (31.2%)	6 (50%)	
**CAD**			0.918			1.000
No	38 (92.7%)	24 (88.9%)		14 (87.5%)	10 (83.3%)	
Yes	3 (7.3%)	3 (11.1%)		2 (12.5%)	2 (16.7%)	
**HLP**			0.411			1.000
**No**	32 (78%)	24 (88.9%)		14 (87.5%)	11 (91.7%)	
**Yes**	9 (22%)	3 (11.1%)		2 (12.5%)	1 (8.3%)	
**FPG, mmol/L (mean** ± **SD)**	7.4 ± 2.9	8.4 ± 4.4	0.284	7.9 ± 3.4	8.7 ± 3.9	0.567
**SBP, mmHg (mean** ± **SD)**	147.8 ± 24.6	153.1 ± 29.0	0.415	149.2 ± 19.3	160.5 ± 33.3	0.270
**DBP, mmHg (mean** ± **SD)**	85.1 ± 14.8	85.1 ± 19.0	0.985	89.4 ± 12.7	95.2 ± 15.4	0.284
**DNT, min (mean** ± **SD)**	36.0 ± 18.6	39.0 ± 22.5	0.557	37.4 ± 33.9	54.6 ± 25.1	0.152
**NIHSS (mean** ± **SD)**	8.9 ± 6.4	14.2 ± 6.3	0.001	8.2 ± 5.7	13.3 ± 5.9	0.029

Abbreviations: ASPECTS, Alberta Stroke Program Early CT Score; LVO, large vessel occlusion; SD, standard deviations.

^a^Continuous variables were presented as mean ± SD, and categorical variables as number (%).

^b^ *p* values were calculated using the Student's *t‐*test for continuous variables and the *χ* ^2^ test or Fisher exact for categorical variables.

In the training set (*N* = 68), 41 patients were classified as having a good prognosis (mRS 0–2) and 27 as having a poor prognosis (mRS 3–6). In the test set (*N* = 28), 16 patients were in the good prognosis group, whereas 12 were in the poor prognosis group. Key findings from the training set included significant differences in ASPECTS scores and NWU (ASPECTS‐NWU) values between the good and poor prognosis groups. Specifically, the good prognosis group had a higher ASPECTS score (9.5 ± 1.1 vs. 8.2 ± 2.1, *p *= 0.005) and ASPECTS‐NWUFCT (2.8 ± 2.0 vs. 5.9 ± 4.6, *p *= 0.002) compared to the poor prognosis group. Notably, the progression of ΔNWU was significantly greater in the poor prognosis group (3.4 ± 4.7 vs. 0.6 ± 2.0, *p *= 0.007). In the test set, the good prognosis group also demonstrated a higher ASPECTS score (9.4 ± 1.0 vs. 8.8 ± 2.8, *p* = 0.435) compared to the poor prognosis group, although this difference did not reach statistical significance. However, the time from symptom onset to imaging was significantly shorter for the good prognosis group (22.7 ± 6.2 vs. 16.6 ± 8.2, *p *= 0.034).

### 3.2 Selection of Risk Factors

To identify significant predictors for poor prognosis in ALVOS patients, we applied LASSO regression, Boruta, and univariable LR for feature selection. Each method contributed to refining the variable set, ultimately enhancing the robustness of the final predictive model. Before applying feature selection, we first explored the relationships among clinical and imaging variables using a correlation matrix (Figure S1). This analysis revealed strong correlations between key variables such as ASPECTS, ASPECTSFCT, and NIHSS, indicating their potential relevance in predicting poor outcomes in ALVOS patients.

**FIGURE 2:**
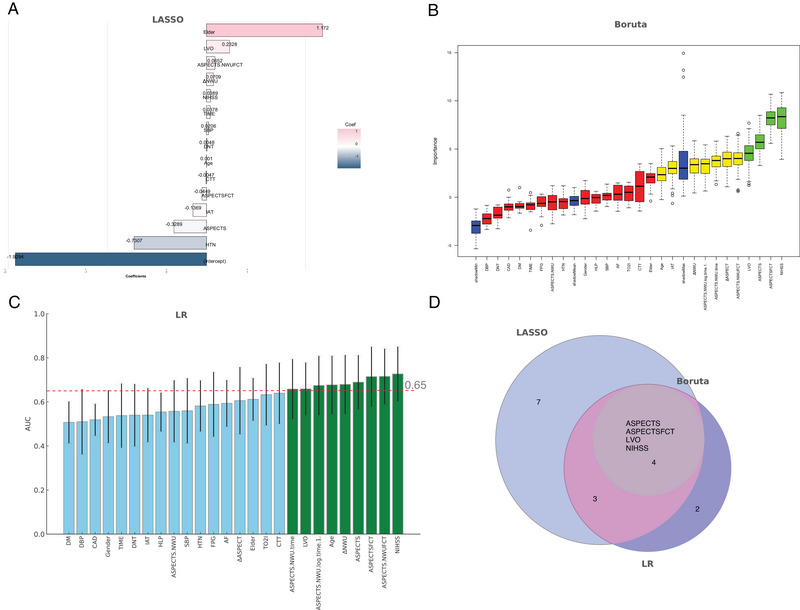
Feature selection and model performance for predicting ALVOS prognosis. (A) LASSO regression analysis for variable selection. (B) Boruta algorithm feature importance ranking, demonstrating the top variables contributing to the predictive model. (C) LR model performance comparison for individual variables, evaluated by AUC values. (D) Venn diagram summarizing overlapping features selected by LASSO, Boruta, and LR. The central overlap includes ASPECTS, ASPECTSFCT, LVO, and NIHSS. ASPECTS, Alberta Stroke Program Early CT Score; AUC, area under the curve; LASSO, Least Absolute Shrinkage and Selection Operator; LR, logistic regression; LVO, large vessel occlusion; NIHSS, National Institutes of Health Stroke Scale; NWU, net water uptake; SD, standard deviations.

Figure 2A illustrates the results of LASSO regression, which systematically reduced the coefficients of less impactful variables to zero as the regularization parameter (lambda) increased, effectively narrowing down the predictor set. This approach highlighted key predictors, such as ASPECTS, ASPECTSFCT, NIHSS, and LVO, as essential contributors to the risk of poor outcomes (Figure S2). Similarly, Figure 2B presents the Boruta algorithm's variable importance ranking, which confirmed the significance of these predictors by iteratively comparing them to randomly permuted shadow variables. This technique reaffirmed the relevance of ASPECTS, ASPECTSFCT, NIHSS, and LVO, indicating their consistent importance across multiple selection criteria (Table S1). Furthermore, Figure 2C displays the results of univariable LR, where odds ratios were calculated for each candidate predictor. On the basis of the results of the univariable LR analysis, several key variables were identified with an AUC greater than 0.65 (Wang et al. 2020), indicating moderate to good discriminatory ability for predicting poor prognosis in ALVOS patients (Table S2).

Finally, Figure 2D summarizes the overlapping results from LASSO, Boruta, and univariable LR in a Venn diagram, showing that ASPECTS, ASPECTSFCT, LVO, and NIHSS were consistently selected as key predictors across all methods. This multi‐method validation provides strong evidence of the predictive power of these variables, supporting their inclusion in the final prognostic model.

### 3.3 Model Development and Validation

The development of a predictive model for poor prognosis in ALVOS patients was guided by the integration of clinical and imaging variables, including LVO status, ASPECTS, ASPECTSFCT, and NIHSS scores. These variables were selected on the basis of their statistical significance and relevance to patient outcomes.

**FIGURE 3:**
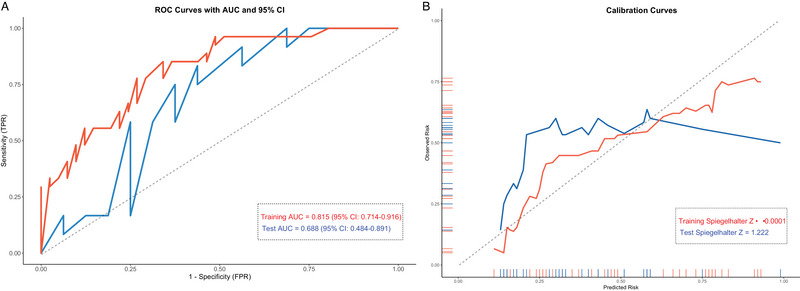
(A) ROC curves for the training and test sets, showing the discriminatory ability of the logistic regression model. (B) Calibration curves for the training and test sets, illustrating the agreement between predicted and observed risk of poor prognosis. AUC, area under the curve; CI, confidence interval; ROC, receiver operating characteristic.

To evaluate the model's performance, we conducted both discrimination and calibration analyses. Figure 3A presents the ROC curves for the training and test datasets, indicating the model's ability to distinguish between good and poor prognostic outcomes. The model achieved an AUC of 0.815 (95% CI: 0.714–0.916) in the training set, demonstrating good discriminatory capability. However, in the test set, the AUC was 0.688 (95% CI: 0.484–0.891), suggesting moderate performance and highlighting the need for further model refinement to improve generalizability. Calibration of the model was assessed using calibration curves, as shown in Figure 3B. In the training set, the model demonstrated excellent calibration (Spiegelhalter *Z* < 0.0001), indicating a strong agreement between predicted and observed outcomes. In contrast, the test set exhibited some calibration issues, with a Spiegelhalter *Z* score of 1.222, suggesting a slight overestimation of risk in certain cases. This finding underscores the importance of additional validation to ensure robust model performance across diverse patient populations.

**FIGURE 4:**
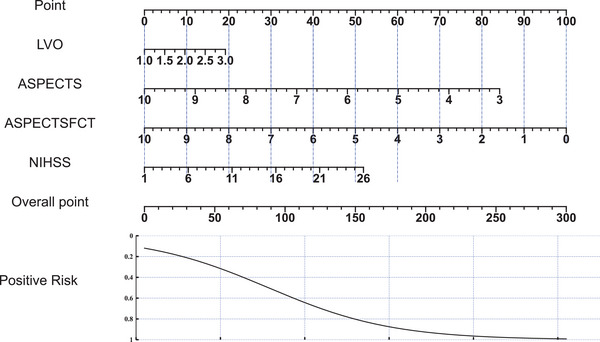
Nomogram for predicting poor prognosis in ALVOS patients. The nomogram developed for individualized risk assessment of poor prognosis in patients with ALVOS incorporates key predictor. LVO, large vessel occlusion; NIHSS, National Institutes of Health Stroke Scale.

A nomogram was constructed to provide an individualized risk assessment for each patient, as shown in Figure 4. This tool assigns points to each predictor based on its contribution to the risk model, with the cumulative score corresponding to the probability of poor prognosis. The nomogram facilitates rapid estimation of a patient's risk by translating the complex LR model into a user‐friendly graphical representation, thus aiding in clinical decision‐making.

## 4 Discussion

This study provides a comprehensive analysis of risk factors associated with poor prognosis in ALVOS patients, utilizing the ASPECTS‐NWU model to enhance prognostic precision. Our findings support the utility of ASPECTS‐NWU, alongside clinical factors such as NIHSS, ASPECTSFCT, and LVO status, in stratifying risk and guiding treatment strategies.

The ASPECTS‐NWU model, which incorporates quantitative imaging metrics, offers a more nuanced understanding of tissue viability and infarct progression than conventional ASPECTS scoring alone. Consistent with recent literature, we found that higher ASPECTS and ASPECTSFCT scores were associated with a better prognosis, whereas increased NWU metrics were linked to poor outcomes, likely due to the indication of substantial edema and infarct size (Ghozy et al. 2024; Ng et al. 2022). This emphasizes the importance of integrating both initial and follow‐up imaging metrics in assessing stroke severity and potential recovery.

Our use of feature selection methods, including LASSO regression, Boruta, and univariable LR, reinforced the significance of variables such as ASPECTS, ASPECTSFCT, NIHSS, and LVO status, with these variables emerging as shared predictors across all methods. This multi‐method validation supports the robustness of these features in predicting ALVOS outcomes, as shown in the final model with an AUC of 0.815 in the training set.

The predictive model demonstrated good discriminatory power and calibration in the training set, as evidenced by the ROC curves and calibration plots. However, the model's performance in the test set was comparatively moderate (AUC = 0.688), suggesting potential limitations in generalizability and robustness. To improve model performance, future research should prioritize expanding the dataset with larger, more diverse patient populations across multiple centers (Srivastava et al. 2022). This would not only enhance statistical power but also improve model adaptability to variations in stroke severity, treatment protocols, and imaging practices. Additionally, although the ASPECTS‐NWU approach provides a more refined imaging‐based metric, it may be limited by variability in CT imaging protocols and HU measurement precision. Future studies should consider standardizing imaging parameters to ensure reproducibility and enhance the model's clinical applicability (Jeon et al. 2023). Establishing harmonized CT acquisition settings and incorporating automated image preprocessing pipelines that apply HU normalization techniques may help reduce inter‐scanner variability. Another promising approach is the use of machine learning techniques to enhance imaging standardization and feature extraction. Deep learning‐based algorithms trained on diverse datasets could be leveraged to automatically adjust for scanner‐specific variations, segment ischemic regions more accurately, and refine NWU quantification.

The constructed nomogram serves as a practical tool for clinicians, facilitating individualized risk assessment by translating complex LR outcomes into an accessible graphical format. For instance, in patients predicted to have a higher risk of poor outcomes, early rehabilitation strategies or neuroprotective interventions could be initiated alongside standard reperfusion therapies, ensuring a more proactive and tailored treatment approach. Furthermore, ASPECTS‐NWU monitoring over time could help refine post‐reperfusion management, identifying patients at higher risk for malignant edema or hemorrhagic transformation who may require early neurosurgical consultation or decompressive hemicraniectomy. In stroke units or resource‐limited settings, where decision‐making must be rapid and precise, integrating ASPECTS‐NWU into automated imaging platforms could facilitate real‐time risk stratification, optimizing patient triage and ICU admission decisions. This aligns with the growing emphasis on personalized medicine in stroke management, where tailored treatment approaches have shown promise in improving patient outcomes (Al‐Maini et al. 2023). By integrating ASPECTS‐NWU‐driven risk assessment into emergency workflows, clinicians can make data‐informed decisions regarding acute interventions, secondary prevention strategies, and long‐term rehabilitation planning, ultimately contributing to more individualized and effective stroke care.

Further research should aim to validate this model in larger, multicenter cohorts, ensuring broader applicability across diverse populations. Integrating advanced imaging modalities like MRI and perfusion CT could provide a more comprehensive assessment of infarct evolution and tissue viability. Diffusion‐weighted imaging and perfusion‐weighted imaging could enhance infarct characterization, particularly when early non‐contrast CT findings are inconclusive. Machine learning approaches could also enhance predictive accuracy by enabling more sophisticated data integration from multimodal sources (Daidone et al. 2024). Convolutional neural networks and radiomics‐based feature extraction may identify subtle imaging patterns overlooked by traditional scoring systems. Future studies should explore hybrid prognostic models that combine ASPECTS‐NWU with perfusion and diffusion imaging features using AI‐driven analytics. By integrating quantitative CT‐based assessments with perfusion‐weighted insights and AI‐driven pattern recognition, stroke prognostication could be further refined to support personalized treatment strategies.

## 5 Conclusions

In conclusion, this study highlights the potential of ASPECTS‐NWU and other clinical imaging factors in predicting ALVOS outcomes. The developed nomogram offers an accessible tool for risk stratification, contributing to personalized stroke management. Further research to validate and refine this model could ultimately enhance prognostic accuracy, guiding treatment decisions and improving patient care in AIS.

## Author Contributions

**Hongru Ou**: conceptualization, data curation, funding acquisition, and writing–original draft. **Huanhua Wu**: conceptualization, methodology, software, investigation, validation, formal analysis, visualization, and funding acquisition. **Shuolong Wu**: methodology, investigation, resources, and data curation. **Qian Cao**: software, formal analysis, data curation, and resources. **Xiaozheng Cao**: investigation and supervision. **Guanye Zhang**: funding acquisition and project administration. **Jiabin Mo**: data curation, formal analysis, and visualization. **Youzhu Hu**: conceptualization, funding acquisition, project administration, and writing–review and editing.

## Ethics Statement

The study was approved by the Ethics Committee of The Affiliated Shunde Hospital of Jinan University, Approval Reference Number [JDSY‐LL‐2023087].

## Conflicts of Interest

The authors declare no conflicts of interest.

### Peer Review

The peer review history for this article is available at https://publons.com/publon/10.1002/brb3.70544

The scientific guarantor of this publication is Dr. Youzhu Hu (Department of General Surgery, The Affiliated Shunde Hospital of Jinan University & Department of Hepatobiliary Surgery, The First Affiliated Hospital, Jinan University).

## Supporting information



Supporting Information

## Data Availability

The data that support the findings of this study are available from the corresponding author, Youzhu Hu, upon reasonable request.
