# Supplementary material for: Risk Factors and Prognostic Models in Acute Large Vessel Occlusion Stroke: Insights From ASPECTS‐Net Water Uptake
Source: Brain Behav. 2025 May 13;15(5):e70544. doi: 10.1002/brb3.70544 (PMC12069852; doi:10.1002/brb3.70544)
Supplement: Supplementary file 1 — Supporting Information [file BRB3-15-e70544-s001.docx]

**Supplementary Materials for Hongru Ou et al.**

**
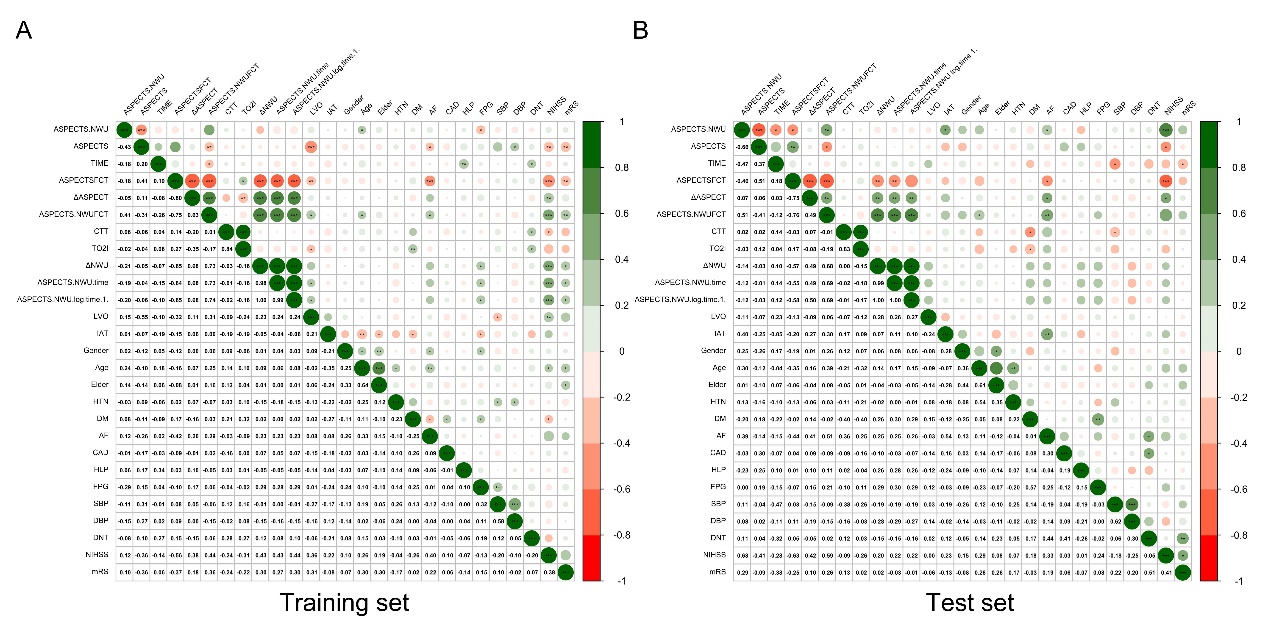
**

***Figure S1.*** Correlation matrix of clinical and imaging variables in training and test sets. Correlation heatmaps depicting relationships among clinical and imaging variables in (A) the training set and (B) the test set. Positive correlations are represented in green, while negative correlations are shown in red, with the intensity of color and size of the circles indicating the strength of the correlation.

**
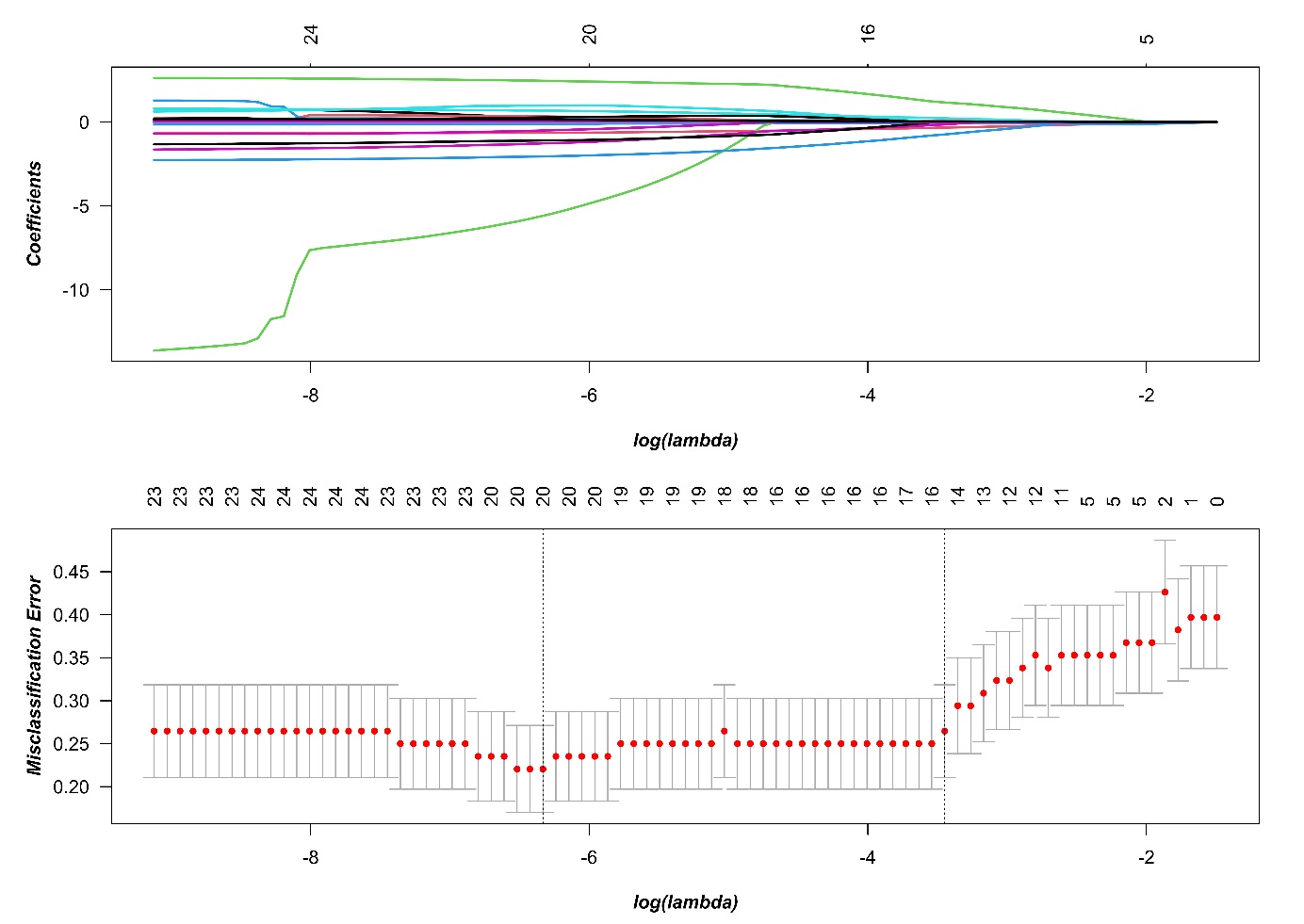
**

***Figure S2.*** LASSO regression path and cross-validation for variable selection. (A) LASSO coefficient path diagram for feature selection. Each line represents the trajectory of a predictor variable’s coefficient as the regularization parameter (lambda) varies. As lambda increases, less influential coefficients shrink toward zero, facilitating the selection of only the most predictive variables. (B) Cross-validation plot for lambda selection. The red dots represent mean misclassification error for each lambda value, with error bars indicating standard deviation. The optimal lambda is chosen where the cross-validated misclassification error is minimized, balancing model complexity and prediction accuracy.

***Table S1.*** Result of Boruta algorithm in the training set.

|  | meanImp | medianImp | minImp | maxImp | normHits | decision |
| --- | --- | --- | --- | --- | --- | --- |
| ASPECTS.NWU | -0.28248 | -0.42899 | -2.27784 | 1.203544 | 0 | Rejected |
| ASPECTS | 5.4949 | 5.417703 | 2.961267 | 10.17381 | 0.777778 | Confirmed |
| TIME | -1.09885 | -1.36838 | -2.28555 | 1.387543 | 0 | Rejected |
| ASPECTSFCT | 7.990319 | 8.012449 | 4.723791 | 9.970333 | 0.969697 | Confirmed |
| ΔASPECT | 4.248832 | 4.465732 | 1.352808 | 7.29966 | 0.575758 | Tentative |
| ASPECTS.NWUFCT | 4.441928 | 4.500722 | 1.870121 | 7.531811 | 0.626263 | Tentative |
| CTT | 0.410662 | 0.290425 | -1.68324 | 2.212848 | 0 | Rejected |
| TO2I | 0.485451 | 0.656601 | -1.09478 | 2.655981 | 0 | Rejected |
| ΔNWU | 3.729926 | 3.80624 | 0.728342 | 6.360849 | 0.535354 | Tentative |
| ASPECTS.NWU.time | 3.446646 | 3.480783 | 0.44328 | 6.768235 | 0.484848 | Tentative |
| ASPECTS.NWU.log.time.1. | 3.41371 | 3.452203 | 0.715237 | 5.955256 | 0.454545 | Tentative |
| LVO | 4.94302 | 5.133621 | 1.37469 | 8.078149 | 0.676768 | Confirmed |
| IAT | 3.58765 | 3.605113 | 1.43652 | 6.490232 | 0.525253 | Tentative |
| Gender | 0.229187 | 0.168696 | -1.33092 | 1.869537 | 0 | Rejected |
| Age | 1.959539 | 1.919676 | -0.82407 | 3.474392 | 0.020202 | Rejected |
| Elder | 1.621548 | 1.333849 | 0.171044 | 3.600324 | 0.020202 | Rejected |
| HTN | -0.58037 | -0.66827 | -2.09354 | 1.203077 | 0 | Rejected |
| DM | -0.42694 | -0.53821 | -2.02436 | 1.001002 | 0 | Rejected |
| AF | -0.27718 | -0.22925 | -1.7483 | 0.887808 | 0 | Rejected |
| CAD | -1.05502 | -0.94033 | -2.03875 | 0.018564 | 0 | Rejected |
| HLP | 0.041444 | 0.050376 | -1.35898 | 2.329021 | 0 | Rejected |
| FPG | -0.6437 | -1.13157 | -1.76426 | 0.975638 | 0 | Rejected |
| SBP | -0.19845 | -0.35237 | -2.10041 | 1.166687 | 0 | Rejected |
| DBP | -2.61522 | -2.78538 | -3.54845 | -0.34097 | 0 | Rejected |
| DNT | -1.38873 | -1.34578 | -2.92624 | 0.694707 | 0 | Rejected |
| NIHSS | 9.013407 | 9.212646 | 3.399592 | 11.717 | 0.959596 | Confirmed |

***Table S2.*** Result of univariable logistic regression in the training set.

| Variable | OR | CI | *p* value | AUC |
| --- | --- | --- | --- | --- |
| ASPECTS.NWU | 1.15 | 0.85-1.54 | 0.37 | 0.557362 |
| ASPECTS | 0.57 | 0.39-0.85 | 0.01 | 0.68925 |
| TIME | 1.03 | 0.97-1.11 | 0.33 | 0.53794 |
| ASPECTSFCT | 0.7 | 0.57-0.86 | <0.001 | 0.714544 |
| ΔASPECT | 1.22 | 1.01-1.49 | 0.04 | 0.605691 |
| ASPECTS.NWUFCT | 1.35 | 1.1-1.64 | <0.001 | 0.715447 |
| CTT | 1 | 0.99-1 | 0.19 | 0.639566 |
| TO2I | 0.74 | 0.47-1.17 | 0.2 | 0.632791 |
| ΔNWU | 1.29 | 1.07-1.56 | 0.01 | 0.679313 |
| ASPECTS.NWU.time | 26.4 | 1.27-550.63 | 0.03 | 0.658085 |
| ASPECTS.NWU.log.time.1. | 1.36 | 1.08-1.71 | 0.01 | 0.674345 |
| LVO2 | 1.32 | 0.11-16.04 | 0.83 | 0.658988 |
| LVO3 | 3.95 | 1.37-11.41 | 0.01 | - |
| IAT1 | 0.72 | 0.27-1.93 | 0.52 | 0.540199 |
| Gender2 | 1.33 | 0.49-3.61 | 0.58 | 0.532972 |
| Age | 1.04 | 1-1.09 | 0.03 | 0.677055 |
| Elder1 | 5.33 | 1.27-22.44 | 0.02 | 0.611563 |
| HTN1 | 0.47 | 0.16-1.34 | 0.16 | 0.581752 |
| DM1 | 1.1 | 0.31-3.92 | 0.88 | 0.507227 |
| AF1 | 2.92 | 0.9-9.48 | 0.08 | 0.593496 |
| CAD1 | 1.58 | 0.3-8.5 | 0.59 | 0.51897 |
| HLP1 | 0.44 | 0.11-1.82 | 0.26 | 0.554201 |
| FPG | 1.09 | 0.94-1.25 | 0.26 | 0.588979 |
| SBP | 1.01 | 0.99-1.03 | 0.41 | 0.559621 |
| DBP | 1 | 0.97-1.03 | 0.99 | 0.509937 |
| DNT | 1.01 | 0.98-1.03 | 0.55 | 0.539747 |
| NIHSS | 1.13 | 1.04-1.23 | <0.001 | 0.726739 |
